# Validation of Rosenberg self-esteem scale among Norwegian adolescents – psychometric properties across samples

**DOI:** 10.1186/s40359-024-02004-0

**Published:** 2024-09-27

**Authors:** U.K Moksnes, G.A Espnes, M.E.B Eilertsen, H.N Bjørnsen, R Ringdal, Gørill Haugan

**Affiliations:** 1 Department of Public Health and Nursing, Norwegian University of Science and Technology, Trondheim, Norway; 2 Faculty of Nursing and Health Science, Nord University, Levanger, Norway; 3 NTNU Social Research, Trondheim, Norway; 4 Department of Child and Adolescent Health Promotion Services, Division for Mental and Physical Health, Norwegian Institute of Public Health, Levanger, Norway

**Keywords:** Rosenberg self-esteem scale, Validity, Factor structure, Psychometric properties

## Abstract

**Background:**

Self-esteem refers to the evaluative and affective dimensions of the self-concept and is important for positive mental health and overall functioning during adolescence. The Rosenberg Self-esteem scale (RSES) is one of the most frequently used and widely accepted instruments assessing self-esteem; however, the psychometric properties of the instrument have not been investigated in a Norwegian adolescent population. The present study’s aim is to investigate the factor structure, construct validity and reliability of the RSES among adolescents 14–21 years.

**Methods:**

The study was based on two cross-sectional samples (*n* = 1,233/ *n* = 1,816) of adolescents from rural and urban areas in Mid-Norway. Concerning the dimensionality of the RSES, two measurement models were tested using Principal Component Analysis and Confirmatory Factor Analysis: a one-factor model and a two-factor-model.

**Results:**

The results show that a two-factor solution of positive and negative aspects of self-esteem representing “perceived personal competence” and “self-value” had the best fit across the two adolescent samples. The RSES also showed high reliability and correlated in expected directions with measures of life satisfaction, stress, and self-efficacy, supporting the convergent validity of the instrument.

**Conclusion:**

The psychometric properties of the RSES need to be further evaluated in Norwegian adolescent populations based on the dimensionality found in the present study; however overall, the results indicate that the instrument is appropriate for assessing self-esteem among Norwegian adolescents.

## Background

Self-esteem is an important component of the self-concept, playing a crucial role for the individual`s mental health, and overall functioning during the life course [1]. Evaluations of self-esteem relates to how people evaluate their personal abilities and attributes or specific domains in life (e.g. success in school or physical appearance) but can also refer to a general overarching evaluation of oneself as a whole; the latter is often referred to as”global self-esteem”. [2, 3]. According to Rosenberg [3], global self-esteem encompasses an individual’s thoughts and feelings regarding their own worth and significance, representing a “global” or “general” evaluation on self-worth. This definition underscores self-esteem as the evaluative and affective dimension of the self-concept, which is subject to various internal and external influences, particularly during the transformative phase of adolescence [4]. Adolescence is clearly a distinct and change-related time in the context of self-esteem, due to the biological, psychological, social, and cognitive changes and transitions occurring during this life phase [4, 5]. Thus, global self-esteem may act as an indicator of how adolescents overall perceive and manage these challenges [4]. The adolescent period now occupies a greater portion of the life course than ever before, due to the earlier onset of puberty, paralleled with a prolonged period of establishing adult roles and responsibilities. The age range between 10 and 19 years has commonly been used to describe the age of adolescence [5]. However, recently, the age range of 10–24 years has been suggested to be more appropriate, reflecting the present understandings of this life phase and is used to define the sample of school students aged 14–21 years old in this study.

Debates within the literature persist regarding whether self-esteem is a stable or changing personal characteristic [4, 6]. While trajectories may differ among individuals, a general trend suggests an increase in self-esteem from adolescence to middle adulthood, reaching a peak around age 50 to 60, followed by a decline in old age [6–8]. Furthermore, distinct trajectories have been observed during adolescence suggesting that self-esteem is a relatively stable, but not an unchangeable personal characterstic, exhibiting significant individual differences and more variability than in other life phases [4, 9, 10].

In terms of gender differences, research findings suggests that males tend to report higher self-esteem than females during adolescence [11]. However, both genders experience an increase in self-esteem from adolescence to midlife, followed by a decline in old age [1, 10]. The dynamic nature of self-esteem underscores its relatively stable yet adaptable nature. The significance of self-esteem is evident in extensive research linking low self-esteem as a vulnerability factor associated with symptoms of depression and anxiety [7, 12]. Conversely, high self-esteem associates with a range of positive outcomes in different life domains including mental health and higher life satisfaction [7, 8, 13] and acts as a protective factor by potentially buffering against negative stressors [14].

Understanding the developmental trends, characteristics, and mechanisms influencing adolescents' self-esteem holds both theoretical and practical importance in fostering their self-development [4]. Therefore, the necessity of valid instruments assessing self-esteem tailored to the current adolescent population becomes evident. The Rosenberg Self-Esteem Scale (RSES) was developed in 1965 and is one of the most widely used measures of global self-esteem [3, 15]. Originally, the RSES is considered a one-dimensional scale, assessing positive and negative feelings about oneself, elaborated from a phenomenological conception of self-esteem that captures the individuals’ global perception of own worth. Although self-esteem is susceptible to different influences, the construct behind the RSES tends to be more of a personal characteristic than an indicator of functional status [16]. The RSES includes ten items on a four-point scale with responses ranging from strongly disagree (1) to strongly agree (4). Half of the items are positively worded; for example, "*On the whole, I am satisfied with myself*", while the other half is worded negatively; for example, "*At times I think I am no good at all*". Total scores range from 10 to 40, with higher scores indicating higher level of self-esteem [3]. The inclusion of five positively and five negatively worded items is to prevent possible response bias, that is, an individual’s tendency to agreeing with statements regardless of their content. It has been debated whether the RSES measures a single factor or if the scale reflects more than one factor. Most research has supported that RSES measures one factor in adult populations [17–19] and in the university student population [20]. Some studies have found support for a two-factor solution mainly based on the five positively and five negatively worded items of self-esteem [16, 21]. Some of the studies in the adult and in student population also provide support for two factors of RSES [22, 23] and two factors reflecting “self-competence” (one’s instrumental and practical value) and “self-value” (one’s sense of intrinsic self-value) [19]. In the adolescent population there are few studies that have validated the RSES, however, support for both a one-factor model [24] and a two-factor model including positive and negative aspects has been found [25, 26]. The psychometric properties of the instrument have to the authors knowledge, not been investigated in the Norwegian adolescent population.

Although the RSES is well-accepted globally, the present psychometric evaluation is important to establish the utility of an assessment tool aimed for use in the current adolescent populations, and to allow recommendations regarding the applicability and broad usefulness of the scale.

The aim of the present study was to test the psychometric properties of the 10-item version of the RSES, using two cross-sectional samples of Norwegian adolescents 14–21 years of age, addressing factor structure, construct validity and reliability, all of which are interrelated measurement properties. Based on theory and previous psychometric validations of the instrument, two hypothesized models were tested: A one-factor model and a two-factor model based on five positively and five negatively worded items. The following three hypotheses were stated:

Hypothesis 1 (H1): The original one-factor model provides a better model fit than the two-factor model.

Hypothesis 2 (H2): The RSES shows good reliability and construct validity.

Hypothesis 3 (H3): The RSES shows good convergent validity with self-efficacy, satisfaction with life and stress.

## Methods

### Participants

The study is based on two cross-sectional samples of adolescents in lower and upper secondary schools in Mid Norway.

#### Sample 1

The sample for this study is derived from survey data collected among adolescents residing in rural and suburban areas across five municipalities in the county of Trøndelag in 2016 in Mid Norway. The data collection has been conducted every fifth year since 1996 where the same municipalities have been invited. The municipalities represent a population where the majority have education at primary or upper-secondary level [27], however, the sample included a higher share of parents with higher education (Table 1). Out of the 1906 students enrolled in the schools, 1282 actively participated in the study, yielding a commendable response rate of 67%. Instances of non-response were primarily caused by students not being present during data collection, non-willingness to participate, or instances where certain classes, for unspecified reasons, were unable to take part. Unfortunately, detailed information on non-responders is not available. Adolescents < 14 or > 20 years (*n* = 49) were excluded, resulting in a final sample of *n* = 1,233 (64%). Among these, 580 (47%) were female, 644 (52.2%) were male, and 9 did not report their gender [28].

**Table 1:** Demographic characteristics of the samples

**Sample 1**		**Sample 2**		
	Total n (%)		Total n (%)	
Gender				
Boys	580 (47.0)		934 (51.5)	
Girls	644 (52.2)		871 (48.0)	
Missing	9 (0.7)		11 (0.5)	
Age				
14–15 years	381 (30.9)	15–16 years	738 (40.7)	
16–17 years	453 (36.7)	17–18 years	928 (51.1)	
18–20 years	399 (32.3)	19–21 years	148 (8.1)	
Missing	0		2 (0.1)	
Family finances				
Bad economy all the time	113 (9.2)		34 (1.9)	
More or less bad economy	243 (19.7)		76 (4.2)	
Neither had bad or good economy	264 (21.4)		406 (22.4)	
More or less good economy	327 (26.5)		580 (31.9)	
Good economy all the time	254 (20.6)		683 (37.6)	
Missing	32 (2.6)		37 (2.0)	
Parents’ education	Mother	Father	Mother	Father
Primary and lower secondary school	37 (3.0)	69 (5.6)	70 (3.9)	91 (5.0)
Upper secondary school	283 (23.0)	366 (29.7)	302 (16.6)	355 (19.5)
University up to 4 years	303 (24.6)	197 (16.0)	452 (24.9)	308 (17.0)
University more than 4 years	221 (17.9)	161 (13.1)	455 (25.1)	453 (24.9)
Unknown	365 (29.6)	393 (31.9)	478 (26.3)	522 (28.7)
Missing	24 (1.9)	47 (3.8)	59 (3.2)	87 (4.8)
Parents’ job status	Mother	Father	Mother	Father
Fulltime job	798 (64.7)	1018 (82.6)	1262 (69.5)	1432 (78.9)
Part-time job	238 (19.3)	86 (7.0)	220 (12.1)	74 (4.1)
Unemployed / on leave	47 (3.8)	28 (2.3)	48 (2.6)	53 (2.9)
Staying at home	83 (6.7)	32 (2.6)	128 (7.0)	49 (2.7)
Other	41 (3.3)	37 (3.0)	88 (4.8)	108 (5.9)
Missing	26 (2.1)	32 (2.6)	70 (3.9)	100 (5.5)
Total	1233 (100)		1816 (100)	

#### Sample 2

The study sample is drawn from a cross-sectional survey conducted among adolescents in an urban area in Mid Norway in 2016. The survey included one private and four public upper-secondary schools; the recruitment of schools was strategic based on that the school health services implemented universal health promoting activities among the students in these schools. The schools included both vocational and general studies which are representative of Norwegian upper secondary schools. The school districts of the included schools are considered similar in sociodemographic terms and represent two of the four school districts in the municipality mainly from suburban areas; one school was in an agricultural area. Of the totally 3,281 students, 2,145 were approached with the survey, and 97.3% (2,087 students) provided valid responses in the survey. The final sample included a total of 1,816 adolescents within the age range of 15–21 years, considered representative of upper-secondary school students. In the sample, 934 were females (51.5%), and 871 were males (48.0%), and 11 (0.5%) did not report their gender [29].

### Procedure

The data collections for this study received approval from the Regional Committee for Medical Research Ethics under the following approval numbers: sample 1—2014/1996 and sample 2—2011/1655. Before initiating data collection, a written information letter was distributed to all students and parents of those aged 15 years or younger. The letter underscored the voluntary and anonymous nature of participation, informed participants of their right to withdraw from the study and assured them that the collected information would be treated with confidentiality.

In compliance with the Health Research Act [30], adolescents and their parents were required to provide written consent when the adolescents were 15 years or younger. For adolescents aged 16 years and older, consent was obtained through their completion of the questionnaire. The data were collected by a hard copy questionnaire consisting of validated and primary recognized scales for use in the Norwegian adolescent population. The administration of questionnaires took place with the assistance of teachers in whole class groups during a regular school session of 45 min in the year 2016. Students who did not want to participate in the study returned a blank questionnaire in an envelope to the teachers, and they were allowed to do other homework.

### Measures

Self-esteem was assessed using Rosenberg's Self-Esteem Scale [3], a 10-item instrument including a four-point scale (ranging from 1—strongly disagree to 4—strongly agree). The scale comprises five positively worded statements (e.g., "On the whole, I am satisfied with myself") and five negatively worded statements (e.g., "I feel I do not have much to be proud of"). The total score, obtained by summing the items, ranges from 10 to 40, with higher scores indicating elevated levels of global self-esteem. The reliability of the scale, measured by Cronbach's alpha, is reported in Table 2.

**Table 2:** Means (M), standard deviation (SD), correlations, Cronabch`s alphas for self-esteem, stress, life satisfaction and self-efficacy in sample 1 (sample 2 in parenthesis)

	Sample 1		Sample 2		Life Satisfaction	Stress	Self-efficacy	Cronbach’s alpha
	Mean (SD)		Mean (SD)					
Self-esteem: 10 items	28.14 (5.51)	727	28.16 (6.40)	1641	.45** (.66**)	-.28 (-.46**)	.42** (.63**)	.81 (.90)
Self-esteem–self-liking 5 items	14.94 (3.03)	720	14.94 (3.22)	1641	.60** (.68**)	-.34 (-.36**)	.53** (.64**)	.87 (.88)
Self-esteem–self-competence 5 items	13.83 (2.62)	720	11.59 (3.87)	1641	.50** (.52**)	-.42** (-.45**)	.39** (.50**)	.87 (.87)
Life satisfaction	24.51 (8.30	1202	21.56 (7.02)	1657		-.40** (-34**)	.45** (.57**)	.66 (.89)
Stress	62.29 (15.50)	730	73.19 (26.70)	1615			-.24** (-.33**)	.94 (.95)
Self-efficacy	29.27 (5.65)	712	30.10 (5.48)	1631				.93 (.93)

^**^significant at the 1% level. Correlations: Sample 1: active sample ranging between *n* = 705 – *n* = 720. Sample 2: effective sample ranging between *n* = 1580 – *n* = 1641

Life Satisfaction (LS) was assessed using the 5-item Satisfaction with Life Scale (SWLS) [31, 32]. This one-dimensional instrument assesses the cognitive aspect of subjective wellbeing through a seven-point Likert scale (ranging from 1—strongly disagree to 7—strongly agree). Examples of items include “In most ways my life is close to my ideal”, “The conditions of my life are excellent” and “I am satisfied with my life”. A higher score, ranging from 5 to 35, signifies higher life satisfaction. The SWLS deemed suitable for assessing LS in both adults and adolescents [31–33], exhibits a reliable internal consistency as indicated by Cronbach's alpha in Table 2.

Stress was measured using the Norwegian 30-item version of the Adolescent Stress Questionnaire (ASQ-N) [34]. This instrument aims to capture normative stressors experienced by adolescents and the degree to which these stressors pose psychological challenges. The ASQ-N consists of seven dimensions and can also be used as a one-dimensional scale. The seven dimensions cover stress related to: school performance (e.g. having to study things you do not understand), school/leisure conflict (e.g. not enough time to have fun), peer pressure (e.g. being hassled for not fitting in), home life (e.g. abiding by petty rules at home), romantic relationships (e.g. Making the relationship work with your boyfriend/girlfriend), teacher/adult interactions (e.g. not being listened to by teachers), and school attendance (e.g. abiding by petty rules at school). Responses are given on a five-point Likert scale (ranging from 1—not at all stressful to 5—very stressful), yielding a total score ranging from 30 to 150. A higher score indicates increased stress levels. The ASQ-N is validated in diverse adolescent samples and demonstrates adequate psychometric properties [35, 36] with the reliability coefficient (Cronbach's alpha) reported in Table 2.

Self-efficacy was assessed using the General Self-Efficacy Scale (GSE), a 10-item one-dimensional instrument rated on a four-point Likert scale (1 = not at all true to 4 = exactly true). Higher sum scores, within the range of 10–40, signify higher level of self-efficacy. Examples of items include “I can always manage to solve difficult problems if I try hard enough”, “I am confident that I could deal efficiently with unexpected events” and “No matter what comes my way, I`m usually able to handle it”. The GSE, validated across different cultural contexts [37], stands as a valid and reliable one-dimensional scale [38, 39] with Cronbach's alpha reported in Table 2.

### Statistical analyses

Descriptive statistics encompassing means and standard deviations were conducted for the scales within the study using IBM SPSS version 27. To conduct psychometric evaluations, an exploratory approach employing Principal Component Analysis (PCA) and Confirmatory Factor analysis (CFA) were undertaken, utilizing Stata 18 (StataCorp 2021). The exploration of the underlying dimensionality of data and the assessment of each item's appropriateness are crucial for establishing a robust measurement model. In this context, both PCA and CFA play pivotal roles, offering distinct information and complementary perspectives on the data [40, 41]. These analyses contribute to a comprehensive understanding of the measurement models employed in the study.

Within the framework of structural equation modeling CFA is specifically designed to address measurement models [42]. One notable strength of CFA lies in its ability to consider random measurement errors, offering a more precise assessment of the psychometric properties of a scale. Through the application of CFA, a high loading of an item signifies a substantial shared variance between the factor and the item. The interpretation of loadings involves a categorization where values below 0.32 are considered poor, those ≥ 0.45 are rated as fair, ≥ 0.55 as good, ≥ 0.63 as very good, and those over 0.71 are deemed excellent [42]. A minimum loading of 0.32, representing approximately 10% overlapping variance with other items in the factor, is considered a reliable benchmark in this evaluation process.

The conventional test of model fit relies on the chi-square statistic (χ^2^) [43]. However, since the chi-square is sensitive to sample size, its interpretation is often enhanced when considered alongside other fit measures. The χ^2^/df serves as an indicator of model fit where values ≤ 2.0 indicate good model fit and values ≤ 3.0 indicate acceptable model fit. Given the non-normal distribution of the data, as evidenced by significant skewness and kurtosis when examining assumptions of multivariate normality, the Satorra-Bentler-scaled chi-square statistic was employed as a goodness-of-fit measure. Additionally, the analysis incorporated the Root Mean Square Error of Approximation (RMSEA) and the Standardized Root Mean Square Residual (SRMS) as fit indices. A good model fit is generally indicated by values ≤ 0.05, and acceptable values in the range ≤ 0.080 or ≤ 0.10 [44]. The Comparative Fit Index (CFI) and the Tucker-Lewis Index (TLI) were used, with an acceptable fit set at 0.90 [44]. Furthermore, to facilitate comparisons among models with varying numbers of factors and items, the Akaike Information Index (AIC) and the Bayesian Information Index (BIC) were recommended [45]. Internal consistency for each dimension in both samples was examined with Cronbach’s alpha. Reliability was further investigated in CFA by means of composite reliability; values ≥ 0.60 are acceptable whereas values ≥ 0.70 are good [44]. The CFA analyses were conducted with listwise deletion of cases using sample 1 as the primary sample and sample 2 as a cross-validating sample. This approach ensures more robustness and generalizability in assessing the measurement models. Construct validity refers to how well an instrument measures the construct it is intended to measure (dimensionality) and is based among others on the construct’s relationships to other variables [45], in this study indicated by convergent validity. Constructs of stress [14], life satisfaction [7, 13], and self-efficacy [46] were selected for assessing convergent validity, using Pearson’s product-moment correlation coefficient analysis. Further, convergent and discriminant validity was assessed by the Average Extracted Value (AVE) as part of the evaluation of the measurement model in CFA.

## Results

### Descriptives

Table 1 presents the descriptive characteristics of the two study samples. Largely, there was an even distribution of gender and age and most adolescents reported that they experienced living in a family with good economy. Further, most adolescents reported that their parents were employed and had higher education (university up to four years or more), however, approximately 30% reported that they did not know about their parents` education. Table 2 presents the means, standard deviations, Cronbach`s alphas and Pearson`s correlation matrices for the scales included in sample 1 and 2 respectively. The mean scores indicated same level of self-esteem and self-efficacy in both samples. The mean scores on life satisfaction were higher in sample 1 than sample 2, whereas the mean scores on stress was higher in sample 2. All scales showed good reliability, except the scale assessing life satisfaction with Cronbach`s alpha 0.66 in sample 1.

### Evaluation of dimensionality of RSES using Confirmatory Factor Analysis (CFA)

To evaluate the dimensionality of the RSES, the one-factor model was tested with CFA termed Model-1. For both sample 1 and sample 2 all factor loadings were significant, with standardized estimates ranging between 0.56-0.80, accompanied by R^2^-values ranging from 0.32 to 0.70. Hence, factor loadings and squared multiple correlations (R^2^) supported the reliability of the items in both samples. However, considering the model fit, all fit indices indicated misspecification of the one-factor model where the chi-square demonstrated an extremely high value, followed by the estimates for RMSEA and all the other fit indices (sample one): χ^2^ = 551.311, (df = 35), χ^2^/df = 15.75, *p* = 0.00001, RMSEA = 0.147 (90% CI, 0.136- 0.158), *p*-value for test of close fit = 0.000001, CFI = 0.85, TLI = 0.81, and SRMR = 0.076. For sample 2 the model fit showed worse fit indices than in sample 1 (Table 3). Overall, all fit indices point to some misspecification of the one-factor measurement model. When evaluating the residuals for both samples, no significant residuals could be seen. However, sample 1 revealed 9 modification indices (MI) higher than 40, while 33 MIs were higher than 10. Some extremely high values were found for several couples of variables, ranging between 40.17 and 96.59. For sample 2 which is large (*N* = 1554), the MI-estimates were even worse (for example: RSES3-RSES4 MI = 310,80; RSES6-RSES9 MI = 221.33). The present data clearly did not fit well to the one-factor model. To be able to identify a model that could fit the data, an exploratory approach was chosen.

**Table 3:** Goodness-of-fit measures for the one-factor and the two-factor model

Sample 1 (Sample 2 in parenthesis)		
Fit Measure	^1^Model-1 1 factor 10 items *n* = 683 (1554)	^2^Model-2 2 factors 10 items *n* = 683 (1554)
χ^2^ *Satorra Bentler*	551.311 (1509.331)	104.524 (302.085)
p-value	< 0.0001	< 0.0001
χ^2^/df ^5^df	15.75 (43.12) 35	3.07 (8.88) 34
RMSEA	0.147 (0.165)	0.055 (0.071)
p-value *(close fit test)*	0.0001	0.226 (0.0001)
SRMR	0.076 (0.081)	0.034 (0.031)
CFI	0.85 (0.83)	0.98 (0.97)
TLI	0.81 (0.78)	0.97 (0.96)
AIC	13,229.962 (32,296.956)	12,785.175 (13,091.709)
BIC	13,365.757 (32,457.413)	12,925.496 (31,257.515)

### Exploring dimensionality of RSES using Principal Component Analysis

**Fig. 1:**
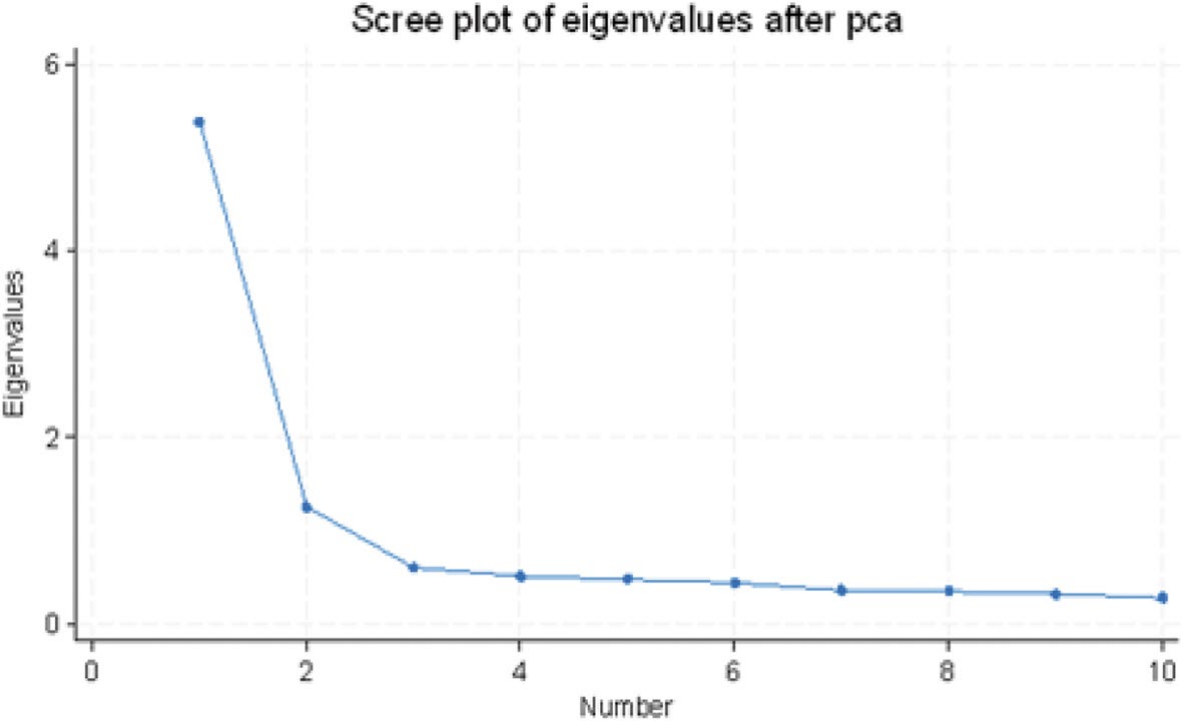
Scree plot of eigenvalues after Principal Component Analysis

Subjecting the RSES to Principal Component Analysis (PCA) in sample 1 the aim was to explain as much of the total variance as possible with as few factors as possible. First, the correlation matrix for the 10 items was checked, followed by a factor test estimating the Kaiser–Meyer–Olkin measure which exceeded the recommended value of 0.60 (0.93), as well as Bartlett’s test of sphericity showing statistical significance (*p* < 0.0001), all of which supported the factorability of the correlation matrix. Running PCA in sample 1, Fig. 1 shows the scree plot of the correlation matrix. As can be seen, two components with Eigenvalue ≥ 1.0 were suggested; component 1 revealed an Eigenvalue of 5.38 accounting for about 54% of the variance while the second component displayed an Eigenvalue of 1.25 accounting for 12.5% of the variance. Since the original RSES is theoretically regarded one-dimensional, we tested the dimensionality in multiple ways. Parallel analysis is regarded as one of the more accurate methods for determining the number of factors or components to retain [47]. When running parallel analysis, the estimates suggested to retain two components (results not shown). Furthermore, when using the maximum likelihood estimation technique, also two factors were suggested. The estimates of AIC and BIC both supported a two-factor structure (Table 3). Hence, the exploratory evaluation clearly pointed toward a two-factor model. Consequently, we searched for the clearest structure by running an oblique Promax rotated solution which theoretically should render a more accurate solution [48]. We used PCA with Eigenvalues ≥ 1 and oblique Promax rotation, explaining 67.5% of the variance (Table 4).

**Table 4:** Principal component analysis with Promax rotation Sample 1

	RSES item	Factor1	Factor2	Uniqueness (error)
Factor 1 – Self-value	RSES1	0.7156	-0.0977	0.3826
	RSES3	0.8412	0.0739	0.3721
	RSES4	0.7963	0.0482	0.4162
	RSES7	0.7132	0.0181	0.5087
	RSES10	0.6377	-0.1646	0.4225
Factor 2 – Self-competence	RSES2	0.0011	0.7691	0.4095
	RSES5	-0.1930	0.5364	0.5332
	RSES6	-0.0239	0.8141	0.3100
	RSES8	0.1236	0.7297	0.5757
	RSES9	-0.0849	0.7964	0.2660

Factor analysis/correlation. Number of observations = 683

Method: iterated principal factors. Retained factors = 2

Rotation: oblique promax (Kaiser on) Number of params = 19

The rotated solution suggested a two-factor model comprising a factor with five positively worded items covering self-value and one factor comprising the five negatively worded items covering self-competence (Table 4). Consequently, we went back to CFA to test this model suggested by PCA. The two-factor model with one positive and one negative dimension of self-esteem revealed a good fit in both samples (Table 3). Composite reliability was 0.089 for the ten items, while the positive and negative self-esteem concepts with five items each both revealed a composite reliability of 0.87. Looking at the Average Variance Extracted (AVE) values, there were no problem with discriminant and convergent validity for both concepts with values of 0.574 and 0.575, respectively (results not shown in table). Table 5 shows the measurement model including factor loadings, t-values, squared multiple correlations (R^2^) and composite reliability for the two-factor-model in both samples, which is the best fitting model in the two samples tested.

**Table 5:** Two-factor measurement model (model 2) for RSES comprising 10 items in sample1 and sample 2 (estimates in parenthesis)

Items	Parameter	Stata Estimate		t-value Sample1(2)	R^2^
Self-value					
Item 1	*λx1,1*	*0.80 (0.81)*		45.94***	*0.64 (0.66)*
Item 3	*λx3,1*	*0.77 (0.79)*		40.74***	*0.59 (0.62)*
Item 4	*λx4,1*	*0.75 (0.72)*		37.21***	*0.56 (0.52)*
Item 7	*λx7,1*	*0.69 (0.71)*		30.37**	*0.48 (0.50)*
Item 10	*λx10,1*	*0.77 (0.81)*		41.10***	*0.60 (0.66)*
Self-competence					
Item 2	*λx2,2*	*0.77 (0.72)*		41.10***	*0.58 (0.51)*
Item 5	*λx5,2*	*0.69 (0.69)*		30.44***	*0.47 (0.48)*
Item 6	*λx6,2*	*0.83 (0.84)*		54.35***	*0.69 (0.71)*
Item 8	*λx8,2*	*0.63 (0.70)*		24.48***	*0.39 (0.50)*
Item 9	*λx9,2*	*0.86 (0.86)*		62.36***	*0.74 (0.73)*
^a^*Composite Reliability for sample 1 (sample 2)*					
Positive self-esteem 5 items		*ρ*_*c*_	*0.87 (0.88)*	*-*	*-*
Negative self-esteem 5 items		*ρ*_*c*_	*0.87 (0.88)*	*-*	*-*

Sample 1 *n* = 683; sample 2 *n* = 1554

^a^ $$\text{Composite Reliability}{\rho c}=\frac{{\left(\sum\uplambda \right)}^{2}}{\left[{\left(\sum\uplambda \right)}^{2} +\sum \left(\uptheta \right)\right]}$$

^***^ *p* < 0.001

### Convergent validity

To evaluate convergent validity, Pearson`s correlation matrices for the original one-factor and the two-factor model of RSES, with self-efficacy, life satisfaction and stress were conducted (Table 2). The results showed significant positive correlations in the expected direction between the one-factor RSES and life satisfaction and self-efficacy and significant negative correlations between RSES and stress. The two-factor model of RSES covering higher levels of “self-value” and “self-competence” also showed significant positive associations with life satisfaction and self-efficacy and significant negative associations with stress. Hence, convergent validity, which among others is based on the construct’s relationship to other variables, was supported for both the one-factor model and the two-factor model. The previously reported AVE-values also supported convergent and discriminant validity only for the two-factor solution.

## Discussion

Self-esteem includes evaluative and affective dimensions of the self-concept and refers to the individual`s overall subjective evaluation of one`s worth as a person and the feeling that one is “good enough” [1]. Self-esteem is likely to be fluctuating and dynamic especially during the formative phase of adolescence and susceptible to internal and external influences. Understanding the development and characteristics of adolescents’ self-esteem has theoretical and practical significance for promoting adolescents’ self-development, mental health and overall functioning [4]. Consequently, the availability of valid instruments for the assessment of self-esteem in the current adolescent population is important [15]. To the author`s knowledge there are few validation studies of the RSES among adolescents, and no previous validation of the RSES has been conducted in the Norwegian adolescent population. Therefore, the novelty of the present study concerns providing knowledge of the psychometric properties of the Norwegian version of the RSES among adolescents between 14–21 years old, using two cross-sectional samples. Three hypotheses (H1-H3) were tested. H1 stated that the original one-factor model provides a better fit than the two-factor model. This hypothesis (H1) did not find support in our study; clearly, the two-factor-model was statistically superior to the one-dimensional solution. Hence, dimensionality of the RSES is questioned. The second hypothesis (H2) concerned the reliability and construct validity of the RSES. The factor loadings were high in both samples, followed by good R^2^-values, indicating that the ten items demonstrated good reliability, explaining a substantial amount of the variance in the self-esteem construct. The internal consistency was also supported by that the Cronbach’s alpha and composite reliability were good. Construct validity involves if the items really assess vital aspects of the self-esteem construct. Based on the factor loadings, the R^2^-values, and the fit indices, we conclude that the ten RSES-items cover essential aspects of self-esteem in this population. Thus, H2 was supported. When testing the convergent validity, both the one-factor model and the two-factor model of RSES demonstrated significant correlations in the expected direction with self-efficacy, life satisfaction and stress. Further, the AVE-values supported both discriminant and convergent validity; hence also H3 found support in the present study.

Concerning model fit, the two-factor-structure disclosed too high estimate for the chi-square in sample 2, however the χ^2^/df was acceptable. Effective sample size was large in sample 2 (*N* = 1554) compared to sample 1 (*N* = 683). As already stated, chi-square is sensitive to sample size; therefore, the χ^2^/df should also be evaluated along with other fit indices [49, 50]. Conversely, the RMSEA estimate has demonstrated lower values with large sample sizes [51]. For an acceptable fit, RMSEA should preferably be ≤ 0.080 or ≤ 0.10 [44], while estimates ≤ 0.050 suggest a good fit. In both samples the RMSEA of the two-factor model was acceptable. Reflecting on the chi-square statistic considering the large sample size (sample 2), the other fit indices were included to assess model fit in both samples, showing acceptable/good estimates. Consequently, the two-factor-model was supported in both samples.

The original one-dimensional measurement model (H1) did not fit well to our data. In both samples, CFA and PCA indicated two substantial factors where the positive items and the negative items loaded on two separate factors. In accordance with our study, previous research has suggested a two-factor solution based in the positively and negatively worded items [16, 21]. Furthermore, some studies among adults and the student population have suggested two RSES-components comprising *self-competence* (one’s instrumental and practical value) and *self-liking/value* (one’s sense of intrinsic self-value) [19]. Thus, the present study supports two factors which can be termed in accordance with Sinclair et al. [19]; (1) ‘self-competence’ and (2) ‘self-value`. Concerning reliability of the items, both concepts (factors) cover aspects of self-esteem.

The five positively worded items involve positive evaluations of one’s personal characteristics, being worthy, having positive attitude towards oneself, all of which cover the construct of ‘self-value` or esteeming oneself. The five negatively worded items cover aspects of not being good at anything, having nothing to be proud of, feeling useless, a failure; these items may be interpreted to embrace ‘perceived personal competence’. Adolescence is a particularly salient period for understanding the development of the self because of the complexity of maturational (puberty), contextual (e.g., school transition), and cognitive (formal operational thinking) changes and transitions occurring during this life phase [1, 4, 9]. Adolescents typically begin to see themselves in a more critical and different way as part of identity development, where one must integrate the more undesirable aspects of the self-concept. Further, youth increasingly seek autonomy from family, along with increased commitment to the peer group and romantic relationships where acceptance, belongingness and external validation is important [52, 53]. Adolescents also experiment and negotiate the peer culture within a continuum of social practices that range from face-to-face relationships to “significant others” in e.g., social media; all of which may impact on adolescents` self-perception [52, 53]. Hence, self-evaluations become increasingly differentiated and complex across roles and relationships during adolescence and cover both aspects of self-value and self-competence.

The third hypothesis investigating the convergent validity of the RSES was supported by showing significant correlations in expected directions. Positive and moderate to strong correlations was found between both the one-factor and two-factor model of RSES and each of life-satisfaction and self-efficacy and negative correlations was found with stress. Interestingly, the correlations were somewhat stronger for the two-factor model, especially with life satisfaction and self-efficacy. The results support previous findings showing that self-esteem is an important factor in association with mental health and quality of life [7, 12, 13] and an important coping resource in association with experience of stress [14]. In reference to the positive association found between self-esteem and self-efficacy, these constructs are found to be theoretically interrelated in that both focus on personal evaluation of the self and contain cognitive (i.e., evaluative), affective, and motivational components [46]. However, the constructs differ in that self-esteem mainly covers the affective evaluation of oneself and that one accepts own strengths and weaknesses in a healthy way, whereas self-efficacy covers the personal and motivational belief in one's ability to achieve goals [46]. Based on the present results, we can conclude that statistically, the dimensionality of the RSES is a two-factor measurement model. We cannot, however, conclude whether this model is based on ‘method effects’ or indicates that the RSES includes two different dimensions among adolescents; that is positive (self-value) and negative (self-competence) self-esteem. The term ‘method effects’ is a well-known issue related to psychological scales which often include both positively and negatively worded items. Method effect refers to variance that is attributable to the “method of measurement” – in this case negative and positive wording of the items – rather than to the construct of interest, often because of response bias, e.g. acquiescent response bias or social desirability bias [54–56]. Nevertheless, several researchers point out that the inclusion of both positively and negatively worded items may introduce new bias; this strategy seems to confound a scale’s factor structure (e.g., negative items load on one or more separate factors) [54, 56]. Our findings align with prior research that questions the inclusion of both positively and negatively worded items, as it can introduce distortions to a scale's factor structure [54, 55]. Addressing the method effect issue, various strategies are proposed to be used, such as utilizing solely positively worded items, maintaining an equal balance of negatively and positively worded items, or incorporating a limited number of negatively worded items solely to mitigate potential response bias—these items would not be used to calculate the overall score [56]. Additionally, some approaches advocate for mixed response options, where half of the items are scored from "strongly disagree" to "strongly agree," while the remaining half are scored inversely. Alternatives include reversing the response options or exclusively incorporating positively worded items to derive a cumulative score [57]. Nevertheless, the anticipated impacts of these strategies should be empirically tested for each scale, emphasizing the need for rigorous empirical validation.

The RSES is a well-established scale and the present study found support for that the two-factor model RSES cover essential aspects of self-esteem. However, further evaluation is required to investigate the psychometric properties of RSES in the adolescent population, also due to the potential method effect. Face validity is an important aspect to consider in the overall evaluation of the psychometric properties of the scale. The items in the RSES could be investigated in focus groups discussion with adolescents and with expert informants in the academic and clinical field. Providing valid evaluation of self-esteem in the adolescent population requires that adolescents can understand and reflect over the relevance, wording, and semantic meaning of the items and that these factors correspond with the language which adolescent use to describe and evaluate their experiences. The age range of the present study samples was wide, and it may be especially challenging for the youngest ones to reflect over some of the items in the same way as the older adolescents. These aspects should be considered in the future evaluation of the RSES in the adolescent population.

### Strengths and limitations

A strength of the study is the inclusion of two large cross-sectional samples of adolescents from rural and urban areas in Mid Norway including an even distribution of sex and age. However, sample 2 was drawn from a relatively homogenous population of mainly suburban Norwegian adolescents; thus, the results may not necessarily be transferable to a more diverse adolescent population. Adequate statistical methods were employed to examine the validity and reliability of the RSES. The effective sample sizes in the analyses differed somewhat (*n* = 683 / *n* = 1554), however, both sample sizes provide strong power to the statistical tests. In the present results two measurement models and their assumptions to the present data have been tested Nevertheless, a good model fit does not guarantee that the “true model” is obtained; other alternative models might fit the data equally well as the identified model [58]. Further, we cannot conclude if the factor solution is caused by data or so-called ‘method effects`. Previous research questions the inclusion of both positively and negatively worded items which may distort a scales’ factor structure [54, 55]. Consequently, the methods effect caused by positively and negatively worded items need to be considered.

## Conclusion

The original one-dimensional model if the RSES-instrument did not fit the present data well. A two-factor model comprising two dimensions which can be interpreted as self-value (the five positively worded items) and self-competence (the five negatively worded items) revealed a good model fit in both samples, supporting the construct validity of the RSES. The two-factor model showed good reliability and convergent as well as discriminant validity. Overall, the results indicate that the RSES instrument is appropriate for assessing self-esteem among Norwegian adolescents. However, based on the two-factor model found, the RSES needs to be further validated in adolescents both in Norway and cross-culturally since few studies have examined the psychometric properties of in adolescent populations.

## Data Availability

The data used in this study are not publicly available. The dataset is available upon reasonable request to the first author. No datasets were generated or analysed during the current study.

## Data availability

No datasets were generated or analysed during the current study.
